# Association Between Diabetic Retinopathy and Carotid Intima-Media Thickness

**DOI:** 10.7759/cureus.15575

**Published:** 2021-06-10

**Authors:** Abdul Subhan Talpur, Zain Amar, Shumaila Zafar, Asadullah Memon, Abdul Habib Eimal Latif, Farukhzad Hafizyar, Sara Hashim, Kefayatullah Nazary

**Affiliations:** 1 Internal Medicine, Liaquat University of Medical and Health Sciences, Jamshoro, PAK; 2 Internal Medicine, Isra University, Hyderabad, PAK; 3 Internal Medicine, Mayo Hospital, Lahore, PAK; 4 Internal Medicine, Kohsar Hospital, Hyderabad, PAK; 5 Internal Medicine, Kabul University of Medical Sciences, Kabul, AFG; 6 Internal Medicine, Ariana Sabet Hospital, Kabul, AFG; 7 Pathology, Bolan Medical College, Quetta, PAK

**Keywords:** diabetes type 2, carotid intima-media thickness, association, diabetic retinopathy, macrovascular complication, microvascular complication

## Abstract

Introduction

Patients with diabetes having advanced stage of diabetic retinopathy (DR) may predict future risk of coronary artery disease. To predict cardiovascular outcomes carotid intima-media thickness (CIMT) is utilized in diabetic patients. The aim of our study was the evaluation of the relationship between retinopathy and CIMT as two valuable non-invasive methods for early detection of micro- and macrovascular complication of diabetes.

Methods

This comparative cross-sectional study was conducted in the internal medicine ward of tertiary care hospital in Pakistan from November 2020 to January 2021. Three hundred patients with type 2 diabetes mellitus and 300 control subjects were enrolled in the study after taking informed consent. Ophthalmological examination was done to screen patients for DR. CIMT was evaluated by a Doppler ultrasound for both carotid arteries.

Results

Carotid artery intimal thickness was more in patients with retinopathy compared to patients without retinopathy in both right (0.77 ± 0.16 vs. 0.66 ± 0.12; p-value: <0.0001) and left carotid artery (0.77 ± 0.15 vs. 0.65 ± 0.11; p-value: <0.0001).

Conclusion

In our study, there was a correlation between DR and CIMT. Screening for DR, which may be a potential early marker for complications, may help detect patients at risk of various macro and microvascular complications.

## Introduction

The prevalence of type 2 diabetes in Pakistan is 16% as per national survey conducted in 2019 [1]. Its prevalence is highest in people aged 51-60 years, those with no formal education, class III obese, and females [1]. Complications in common in diabetes and are responsible for significant morbidity and mortality [2]. The long-term complications of diabetes are commonly divided into micro- and macrovascular complications [3]. Small blood vessels are affected under microvascular complications leading to neuropathy, nephropathy, and retinopathy, whereas the microvascular complications comprise cardiovascular disease, stroke, and peripheral artery disease [4].

Amongst the diabetic population of Pakistan, the prevalence of diabetic retinopathy (DR) is about 26% [5]. Generalized atherosclerosis is another complication of diabetes; it is manifested in the form of ischemic heart disease, cerebrovascular accident, or peripheral vascular disease. Measurement of the common or internal carotid intima-media thickness (CIMT) can be used for the recognition of diabetic atherosclerosis. The CIMT has been already employed for the prediction of cardiovascular outcomes in diabetic patients [6]. Patients with diabetes having advanced stage of DR, with no history of clinical cardiovascular disease (CVD), were found to have higher prevalence of subclinical coronary artery disease. During the early stages of diabetes, the presence of retinopathy may signal a more vigilant cardiovascular assessment, as DR was expressed as the marker for subclinical atherosclerosis [7].

The purpose of our study was to evaluate the relationship between retinopathy and CIMT as two significant non-invasive methods for the early detection of long-term complications of diabetes.

## Materials and methods

This comparative cross-sectional study was conducted in the internal medicine ward of a tertiary care hospital in Pakistan from November 2020 to January 2021. After taking informed consent, 300 patients with type 2 diabetes mellitus were enrolled in the study. Patients were enrolled via consecutive convenient non-probability sampling technique. Patients with age-related macular degeneration, cataract, glaucoma and ocular diseases other than diabetic retinopathy were excluded from the study.

After inclusion, patients’ detailed history was taken to gather data related to their age, gender, hypertension, and hypercholesterolemia status. Blood was drawn via phlebotomy, and sent to the laboratory to test for glycated hemoglobin (HbA1C) and random blood glucose levels. Ophthalmological examination was done to screen the patients for DR via fundoscopy. CIMT was evaluated by a Doppler ultrasound for both carotid arteries. CIMT was calculated as the linear distance between the lumen-intima interface and the media-adventitia interface.

Statistical analysis was done using Statistical Packages for Social Sciences (SPSS) v. 23.0 (IBM Corporation, Armonk, NY, United States). Continuous variables including age, body mass index (BMI) (kg/m^2^), duration of diabetes, and CIMT were analyzed via descriptive statistics and presented as mean and standard deviation (SD); while categorical variables, including gender, hypertension, and hypercholesterolemia status were presented by percentages and frequencies. CIMT between two groups was compared using an independent t-test. A p-value of less than 0.05 meant that there is a difference between the two groups and the null hypothesis is void.

## Results

Out of 300 patients, 141 (47.0%) patients had retinopathy. Patients with retinopathy had longer duration of diabetes (10 ± 6 years vs. 7 ± 5 years; p-value: <0.0001) and higher HbA1C (8.2 ± 1.2% vs. 7.9 ± 1.0%; p-value: 0.01) (Table 1).

**Table 1:** Characteristics of the participants. BMI: body mass index; DR: diabetic retinopathy; HbA1C: glycated hemoglobin; SD: standard deviation.

Characteristics	Patients with DR (n = 141)	Patients without DR (n = 159)	p-value
Age in years (mean ± SD)	53 ± 10	51 ± 10	0.08
Male (%)	81 (57.4%)	90 (56.6%)	0.88
Hypertension (%)	70 (49.6%)	78 (49.0%)	0.9
Hypercholesterolemia (%)	72 (51.0%)	80 (50.3%)	0.9
Duration of diabetes (in years)	10 ± 6 0.0001	7 ± 5	<0.0001
BMI, kg/m^2^	25.2 ± 4.4	24.9 ± 4.3	0.55
Random blood glucose (mg/dL)	242.2 ± 94.2	251.2 ± 89.1	0.3
HbA1C%	8.2 ± 1.2	7.9 ± 1.0	0.01

Intimal thickness was more in patients with retinopathy compared to the patients without retinopathy in both right (0.77 ± 0.16 vs. 0.66 ± 0.12; p-value: <0.0001) and left carotid artery (0.77 ± 0.15 vs. 0.65 ± 0.11; p-value: <0.0001); however, there was no difference carotid artery intimal thickness between right and left carotid artery in patients with diabetic retinopathy. Similar, there was no difference between male and female carotid artery thickness within patients with retinopathy (Table 2).

**Table 2:** Comparison of intimal thickness in patients with and without retinopathy. *Calculated by comparing two groups within patients with retinopathy. **Calculated by comparing patients with retinopathy and patients without retinopathy.

Stratification	Carotid artery intimal thickness (mm)	Patients with retinopathy (n = 141)	Patients without retinopathy (n = 159)	p-value*	p-value**
Location	Right carotid artery	0.77 ± 0.16	0.66 ± 0.12	1.00	<0.0001
	Left carotid artery	0.77 ± 0.15	0.65 ± 0.11		<0.0001
Gender	Male	0.76 ± 0.15	0.65 ± 0.12	0.57	<0.0001
	Female	0.77 ± 0.15	0.65 ± 0.11		<0.0001

## Discussion

Our study had enrolled 300 diabetic participants, out of which 47% showed a prevalence of retinopathy. This association of type 2 diabetes and DR has also been demonstrated in another study conducted by Yau et al., which also suggested no gender-related difference in the trends [7]. In our study, there were more male participants compared to female; however, the ratio was similar between both groups. Furthermore, our study did not find a significant link between BMI and CIMT, which is in accordance with a study conducted by Momeni et al. [8]. Whereas, another study proved to show a significant correlation between maximum BMI and retinopathy [9]. The most plausible explanation of the differences in these results could be the drastic difference in the sample sizes. Another main finding of our study was that HbA1c and retinopathy were positively linked. This finding is backed up by a multicentre Iranian study carried out by Penno et al. [10]. Penno et al. also stated other factors responsible for retinopathy such as younger age, lower age at diabetes diagnosis, shorter diabetes duration, higher value and variability of glycated hemoglobin [10]

The prevalence of retinopathy was found to be significantly associated with a prolonged history of diabetes. The results were consistent in several studies that also proved the connection between longer disease duration and retinopathy [7,11]. Moreover, it was proved that the left and right carotid arteries of patients with retinopathy had a thicker intima compared to those who did not have retinopathy. Several studies have proved this link between CIMT and retinopathy; one of them being conducted by Miyamoto et al. in which 102 patients with diabetes were examined to demonstrate a positive correlation between retinopathy and thickness of the common carotid artery [12]. CIMT is labeled as a surrogate marker of subclinical atherosclerosis, and studies have proved that it helps to identify the likelihood of underlying cardiovascular pathology in patients at a higher risk [6,13-15]. Previously, in a longitudinal study and two recent meta-analyses from population-based cohorts, it has been demonstrated that CIMT is a powerful predictor of negative cardiovascular results [16-20]. Moreover, in population-based studies, CIMT has been proved to be correlated with the development of albuminuria chronic kidney disease [21,22].

To the best of our knowledge, this is the first study in regional setting to study the association between diabetic retinopathy and CIMT. However, since the study was conducted in a single institute and was case-control, further large-scale prospective multi-centric trials are needed to confirm the association. Considering the positive correlation between CIMT and DR, this study suggests that proper screening in patients with retinopathy could help in early diagnoses. This would further help to avoid complications that tag along with CIMT. However, future studies with a larger sample size are required to explore the complications associated with CIMT.

## Conclusions

In this study, we found that diabetic retinopathy is an indicator of CIMT. In diabetic patients, CIMT is known to be a potential cause of several complications. Diabetic patients should be regularly screened for diabetic retinopathy. Moreover, patients who have retinopathy should get themselves checked at regular intervals for CIMT and cardiovascular diseases.

## References

[1] Aamir AH, Ul-Haq Z, Mahar SA (2019). Diabetes Prevalence Survey of Pakistan (DPS-PAK): prevalence of type 2 diabetes mellitus and prediabetes using HbA1c: a population-based survey from Pakistan. BMJ Open.

[2] Papatheodorou K, Banach M, Bekiari E, Rizzo M, Edmonds M (2018). Complications of diabetes 2017. J Diabetes Res.

[3] Deshpande AD, Harris-Hayes M, Schootman M (2008). Epidemiology of diabetes and diabetes-related complications. Phys Ther.

[4] Tuttolomondo A, Maida C, Pinto A (2015). Diabetic foot syndrome as a possible cardiovascular marker in diabetic patients. J Diabetes Res.

[5] Mumtaz SN, Fahim MF, Arslan M, Shaikh SA, Kazi U, Memon MS (2018). Prevalence of diabetic retinopathy in Pakistan: a systematic review. Pak J Med Sci.

[6] Polak JF, Pencina MJ, Pencina KM, O'Donnell CJ, Wolf PA, D'Agostino RB Sr (2011). Carotid-wall intima-media thickness and cardiovascular events. N Engl J Med.

[7] Yau JW, Rogers SL, Kawasaki R (2012). Global prevalence and major risk factors of diabetic retinopathy. Diabetes Care.

[8] Momeni A, Dyani MA, Ebrahimi E, Sedehi M, Naderi A (2015). Association of retinopathy and intima media thickness of common carotid artery in type 2 diabetic patients. J Res Med Sci.

[9] Ogawa K, Ueda K, Sasaki H (2004). History of obesity as a risk factor for both carotid atherosclerosis and microangiopathy. Diabetes Res Clin Pract.

[10] Penno G, Solini A, Bonora E (2013). HbA1c variability as an independent correlate of nephropathy, but not retinopathy, in patients with type 2 diabetes: the Renal Insufficiency And Cardiovascular Events (RIACE) Italian multicenter study. Diabetes Care.

[11] Ding J, Wong TY (2012). Current epidemiology of diabetic retinopathy and diabetic macular edema. Curr Diab Rep.

[12] Miyamoto M, Kotani K, Okada K, Fujii Y, Konno K, Ishibashi S, Taniguchi N (2012). The correlation of common carotid arterial diameter with atherosclerosis and diabetic retinopathy in patients with type 2 diabetes mellitus. Acta Diabetol.

[13] Baldassarre D, Hamsten A, Veglia F (2012). Measurements of carotid intima-media thickness and of interadventitia common carotid diameter improve prediction of cardiovascular events: results of the IMPROVE (Carotid Intima Media Thickness [IMT] and IMT-Progression as Predictors of Vascular Events in a High Risk European Population) study. J Am Coll Cardiol.

[14] Den Ruijter HM, Peters SA, Anderson TJ (2012). Common carotid intima-media thickness measurements in cardiovascular risk prediction: a meta-analysis. JAMA.

[15] Lorenz MW, Markus HS, Bots ML, Rosvall M, Sitzer M (2007). Prediction of clinical cardiovascular events with carotid intima-media thickness: a systematic review and meta-analysis. Circulation.

[16] Yoshida M, Mita T, Yamamoto R (2012). Combination of the Framingham risk score and carotid intima-media thickness improves the prediction of cardiovascular events in patients with type 2 diabetes. Diabetes Care.

[17] Malik S, Budoff MJ, Katz R (2011). Impact of subclinical atherosclerosis on cardiovascular disease events in individuals with metabolic syndrome and diabetes: the multi-ethnic study of atherosclerosis. Diabetes Care.

[18] Katakami N, Mita T, Gosho M (2018). Clinical utility of carotid ultrasonography in the prediction of cardiovascular events in patients with diabetes: a combined analysis of data obtained in five longitudinal studies. J Atheroscler Thromb.

[19] den Ruijter HM, Peters SA, Groenewegen KA (2013). Common carotid intima-media thickness does not add to Framingham risk score in individuals with diabetes mellitus: the USE-IMT initiative. Diabetologia.

[20] Lorenz MW, Price JF, Robertson C (2015). Carotid intima-media thickness progression and risk of vascular events in people with diabetes: results from the PROG-IMT collaboration. Diabetes Care.

[21] Yu Z, Schneck M, Jacobs DR Jr (2011). Association of carotid intima-media thickness with progression of urine albumin-creatinine ratios in The Multi-Ethnic Study of Atherosclerosis (MESA). Am J Kidney Dis.

[22] Shimizu M, Furusyo N, Mitsumoto F (2015). Subclinical carotid atherosclerosis and triglycerides predict the incidence of chronic kidney disease in the Japanese general population: results from the Kyushu and Okinawa Population Study (KOPS). Atherosclerosis.

